# Development of a Subjective Visual Vertical Test System Using a Smartphone With Virtual Reality Goggles for Screening of Otolithic Dysfunction: Observational Study

**DOI:** 10.2196/53642

**Published:** 2024-06-04

**Authors:** Akiko Umibe, Hiroaki Fushiki, Reiko Tsunoda, Tatsuaki Kuroda, Kazuhiro Kuroda, Yasuhiro Tanaka

**Affiliations:** 1 Department of Otorhinolaryngology, Head and Neck Surgery Dokkyo Medical University Saitama Medical Center Koshigaya-shi, Saitama Japan; 2 Department of Otolaryngology Mejiro University Ear Institute Clinic Saitama-shi, Saitama Japan; 3 Kuroda Ear, Nose and Throat Clinic Yatsushiro-shi, Kumamoto Japan

**Keywords:** vestibular function tests, telemedicine, smartphone, virtual reality, otolith dysfunction screening tool, vestibular evoked myogenic potential, iPhone, mobile phone

## Abstract

**Background:**

The subjective visual vertical (SVV) test can evaluate otolith function and spatial awareness and is performed in dedicated vertigo centers using specialized equipment; however, it is not otherwise widely used because of the specific equipment and space requirements. An SVV test smartphone app was developed to easily perform assessments in outpatient facilities.

**Objective:**

This study aimed to verify whether the SVV test smartphone app with commercially available virtual reality goggles can be used in a clinical setting.

**Methods:**

The reference range was calculated for 15 healthy participants. We included 14 adult patients with unilateral vestibular neuritis, sudden sensorineural hearing loss with vertigo, and Meniere disease and investigated the correlation between the SVV test results and vestibular evoked myogenic potential (VEMP) results.

**Results:**

The SVV reference range of healthy participants for the sitting front-facing position was small, ranging from –2.6º to 2.3º. Among the 14 patients, 6 (43%) exceeded the reference range for healthy participants. The SVV of patients with vestibular neuritis and sudden sensorineural hearing loss tended to deviate to the affected side. A total of 9 (64%) had abnormal cervical VEMP (cVEMP) values and 6 (43%) had abnormal ocular VEMP (oVEMP) values. No significant difference was found between the presence or absence of abnormal SVV values and the presence or absence of abnormal cVEMP and oVEMP values; however, the odds ratios (ORs) suggested a higher likelihood of abnormal SVV values among those with abnormal cVEMP and oVEMP responses (OR 2.40, 95% CI 0.18-32.88; *P*>.99; and OR 2, 95% CI 0.90-4.45; *P*=.46, respectively).

**Conclusions:**

The SVV app can be used anywhere and in a short period while reducing directional bias by using virtual reality goggles, thus making it highly versatile and useful as a practical otolith dysfunction screening tool.

## Introduction

### Background

The subjective visual vertical (SVV) test measures a person’s perception of verticality and is performed by placing a fluorescent bar in front of the eyes in a darkened room. It can evaluate otolith function and spatial awareness; however, it is not widely used in outpatient settings because of the equipment and space requirements. SVV tests are generally performed in dedicated vertigo centers using specialized equipment. Vestibular evoked myogenic potential (VEMP) is commonly used for otolith dysfunction testing, but because VEMP amplitude is related to the degree of the sternocleidomastoid muscle tone, it may be impossible to perform the test in patients who are unable to assume a cervical flexion position.

### Study

In recent years, the use of tests and diagnostic tools with smartphone apps has advanced in clinical practice. According to a review conducted in 2017, the most common otolaryngology/head and neck surgery apps used by patients were intended for hearing, tinnitus treatment, and sound measurement [1]. Recently, several SVV tests performed using smartphone apps and virtual reality (VR) goggles have been developed [2-4]. The SVV test using conventional apps is very useful for screening purposes; however, directional bias occurs when the test is conducted in a bright place and it is difficult to make accurate assessments when the body position changes. In this study, we aimed to develop a smartphone app dedicated to SVV evaluation and verify whether the SVV test can be easily performed in outpatient facilities with commercially available VR goggles.

In addition, we hypothesized that if cervical VEMP (cVEMP), ocular VEMP (oVEMP), and SVV are measured in patients with vestibular disorders, some relationship may be observed between c/oVEM*P* values and SVV values. This study may contribute to the development of new diagnostic tools for dizziness.

## Methods

### Overview

**Figure 1:**
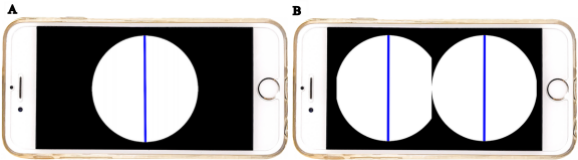
App screens for (A) 2D and (B) virtual reality.

We developed an app called Subjective Visual Vertical, which can be downloaded for free from the Apple Store for Education and Research. This app uses accelerometer values from the iPhone’s motion manager to calculate the angle, measure the absolute vertical position, tilt angle, and SVV, and automatically calculate their average and SD values. This app supports both 2D and VR functions (Figure 1A,B). For the VR goggles, we used a commercially available product (Dasimon) that is compatible with smartphones, and the focal length and the widths of the left and right eyes can be adjusted. In addition, a remote control with a Bluetooth connection was used to operate the app (Elecom VR controller; Elecom Co Ltd).

An iPhone 8 (Apple Inc) was used for this purpose. When the app started (it can be used without an internet connection), the straight bar tilted left and right. When the user thought that the bar was vertical, the remote control was pressed to make the bar still. The mean and SD values of SVV were automatically calculated and recorded in the app. The position of the SVV bar was evaluated as positive when the bar stopped in the clockwise direction relative to the absolute vertical position and as negative when it stopped in the counterclockwise direction.

**Figure 2:**
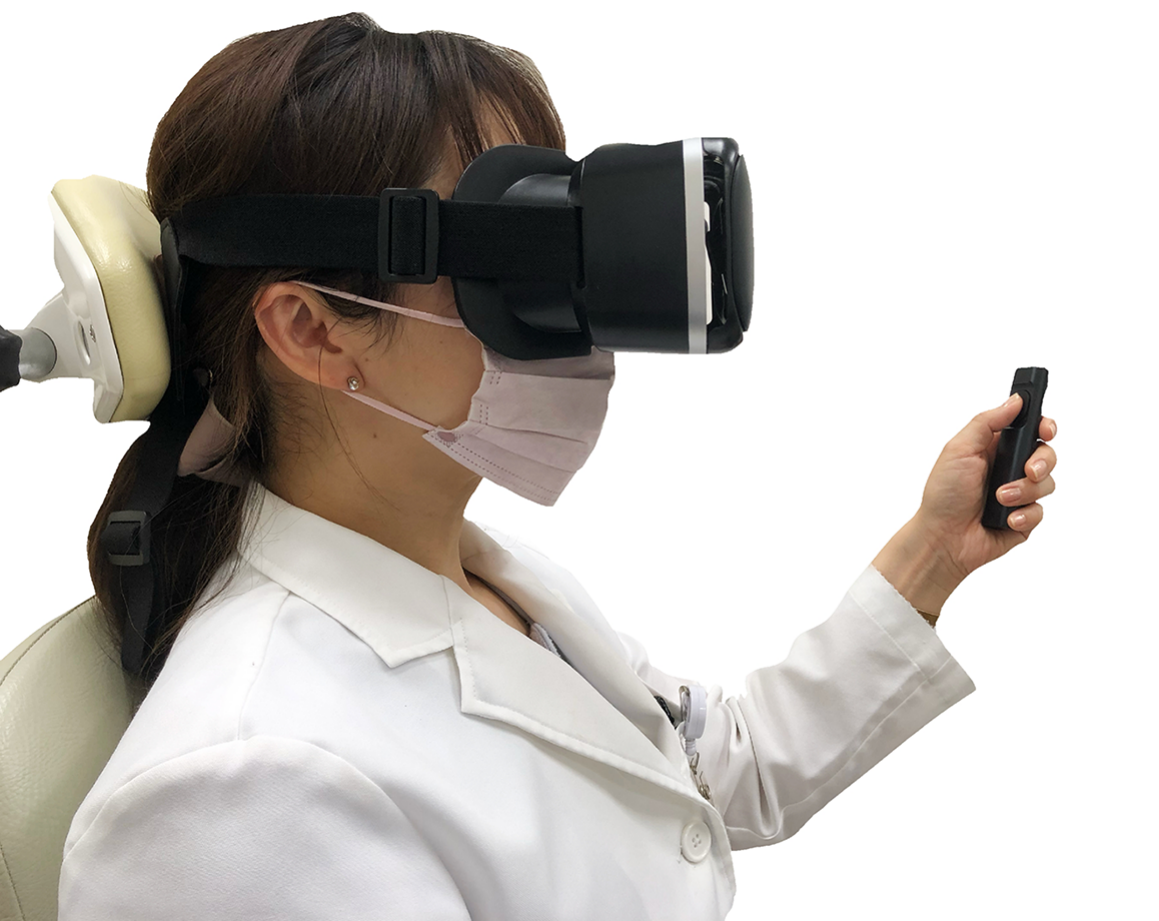
Wearing the virtual reality goggles and operation of the remote control.

The actual measurement method was performed in the following order: (1) before the participants wore goggles, the operation of the app and the intention of the test were explained; (2) the smartphone was connected to the VR goggles worn by the examinee while the app was running (Figure 2); (3) the examinee performed a practice test; and (4) the actual test was performed in sitting positions. The time required was approximately 5-10 minutes per participant (including the explanation).

### Participants

This method was first used with 15 healthy participants (5 male participants and 10 female participants; median age 32, IQR 26-42 years). The detected SVV values were used to calculate the normal range. None of the healthy participants had a history of peripheral or central vestibular disorders. Then, 14 adult patients with unilateral vestibular neuritis (1 male participant, 2 female participants; median age 68, IQR 40-72 years), Meniere disease (4 male participants, 3 female participants; median age 64, IQR 57-79 years), or sudden sensorineural hearing loss with vertigo (4 female participants; median age 63, IQR 49.75-73.25 years) were tested to determine the SVV values. The SVV values were measured and compared with the results of the vestibular function tests, including VEMP, as well as Dizziness Handicap Inventory (DHI) scores.

The exclusion criteria were (1) being younger than 20 years of age, (2) being unable to maintain a sitting or lying position, (3) having central vertigo (stroke and neoplastic lesions), and (4) having traumatic unilateral vestibular disorders.

### Statistical Analyses

G*Power (Institut für Psychologie, Christian-Albrechts-Universität Kiel) was used to calculate the sample size [5]. The effect size was set at *P*=.5, the α error was .05, and the power was 0.8. The required sample size was calculated to be 26; therefore, a sample size of at least 13 participants per group was required.

All statistical analyses were performed using SPSS (version 28; IBM Corp). *P*<.049 was considered statistically significant.

The quantitative continuous variables present in the study were subjected to normality tests like Kolmogrov-Smirnov and Shapiro-Wilk tests. The results of normality tests are illustrated in Multimedia Appendix 1. The variables age and DHI were found to be in normal distribution and cVEMP, oVEMP, and SVV value were found to be in nonnormal distributions. Parametric tests were applied for normally distributed data and nonparametric tests were applied for nonnormally distributed data. The correlation between SVV and the age of healthy participants was done using the Spearman correlation test. The association between SVV and the sex of healthy participants was explored using the Mann-Whitney *U* test. The association between SVV values and cVEMP, as well as oVEMP, were done using the Fisher exact test.

### Ethical Considerations

This study was approved by the clinical research ethics review committee of Dokkyo Medical University Saitama Medical Center (21020), and was performed in line with the principles of the Declaration of Helsinki. The physician conducting the study explained the research and obtained consent from all participants. Each participant was assigned a unique anonymous identifier to protect their privacy.

## Results

### Healthy Participant Data

Table 1 presents SVV data for healthy participants across various head positions. The mean SVV value was 0.21º (SD1.86) with a 95% CI ranging from –0.82 to 1.25. The SE was calculated to be 0.48, indicating the precision of the mean estimate. The median SVV value was 0.71º (IQR —1.44 to 1.22), suggesting a central tendency slightly higher than the mean. The minimum SVV recorded was –2.87º, while the maximum SVV was 3.23º. The reference range was calculated to be –2.6º to 3.23º and included a median value. The range of SVV values spans 6.10 degrees, indicating considerable variability in participants’ perceptions of verticality.

**Table 1:** SVV^a^ data among healthy participants (n=15).

Characteristics		Values
**Age (years), n (%)**		
	20-29	5 (33)
	30-39	6 (40)
	40-49	4 (27)
**Sex, n (%)**		
	Male	5 (33)
	Female	10 (67)
**SVV (degrees)**		
	Mean (95% CI)	0.21 (–0.82 to 1.25, SD1.86)
	SE	0.48
	Reference range	–2.6 to 2.3
	Median	0.71 (—1.44 to 1.22)
	Minimum-maximum	–2.87 to 3.23

^a^SVV: subjective visual vertical.

In the correlation between SVV and the age of healthy participants, the Spearman correlation coefficient (ρ) between SVV and age was –0.045, indicating a very weak negative correlation between these variables. The associated *P* value was .87, suggesting that this correlation was not statistically significant.

In the association between SVV and the sex of healthy participants, for male participants, the mean SVV was 0.38º (SD 1.20º), while the median SVV was 0.69º (IQR –0.81 to 1.40). The *U* statistic, a measure of the difference between the 2 groups, was 23.00. The *P* value for this comparison was .86, indicating no statistically significant difference in SVV between male and female participants. For female participants, the mean SVV was 0.13º (SD 2.17º), and the median SVVº was 0.78 (IQR –1.95 to 1.65).

### Patient Data

All patient data are shown in Table 2. Among those diagnosed with vestibular neuritis, patients’ ages ranged from 40 to 72 years, with both genders represented. SVV values varied from –5.00º to 0.20º, with spontaneous nystagmus observed in all cases. Regarding sudden sensorineural hearing loss, patients were predominantly female and aged between 46 and 76 years, with SVV values ranging from –0.24º to 16.76º. Patients with Meniere disease, primarily female and aged between 47 and 84 years, exhibited SVV values ranging from –5.30º to 5.03º. Diagnostic tests such as cVEMP and oVEMP were administered, with variable results across patients. For VEMP, the side with a decreased response is noted in ] Table 2. Additionally, DHI scores ranged from 2 to 88, indicating varying degrees of functional impairment due to dizziness. Outliers are identified in the table with footnotes, indicating the data points that deviated significantly from the norm within the data set.

**Table 2:** List of all patient data (n=14).

Disease and age (years)			Side		Sex		SVV^a^ value (degrees)		Spontaneous nystagmus		cVEMP^b^	oVEMP^c^		DHI^d^ score
**Vestibular neuritis**														
	40	R^e^		F^f^		0.20		+		N^g^		R	76	
	68	L^h^		F		–5.00^i^		+		N		L	28	
	72	L		M^d^		–2.52^i^		+		N/A^j^		N/A	2	
**Sudden sensorineural hearing loss**														
	46	R		F		2.29		–		N		N/A	64	
	61	R		F		9.39^i^		–		R		R	N/A	
	76	R		F		–0.24		–		B^k^		B	10	
	65	R		F		16.76^i^		+		R		R	74	
**Meniere disease**														
	57	R		F		0.19		–		R		N/A	14	
	47	R		F		1.39		–		R		B	4	
	84	R		F		–5.30^i^		–		B		N/A	40	
	58	L		M		–0.29		–		N		N/A	88	
	64	L		M		–1.40		–		L		R	38	
	79	L		M		5.03^i^		–		L		N/A	12	
	66	L		M		2.19		–		R		N	26	

^a^SVV: subjective visual vertical.

^b^cVEMP: cervical vestibular evoked myogenic potential.

^c^oVEMP: ocular vestibular evoked myogenic potential.

^d^DHI: Dizziness Handicap Inventory.

^e^R: right.

^f^F: female.

^g^N: normal.

^h^L: left.

^i^outlier.

^j^N/A: not applicable.

^k^B: bilateral.

### SVV Values

Table 3 presents the association between SVV values and cVEMP and oVEMP results. Among the 14 patients, 6 (43%) had abnormal SVV values. Among the 14 patients, 9 (64%) had abnormal cVEM*P* values, 6 (43%) had abnormal oVEM*P* values, and 12 (86%) had abnormal SVV or VEM*P* values.

**Table 3:** Association between SVV^a^ with cVEMP^b^ and oVEMP^c^ (N=14).

Variable		SVV value (degrees)			*P* value		OR^d^ (95% CI)
		Normal, n (%)	Abnormal, n (%)				
**cVEMP**					>.99		2.40 (0.18-32.88)
	Normal	3 (38)	1 (20)				
	Abnormal	5 (63)	4 (80)				
**oVEMP**					.46		2.00 (0.90-4.45)
	Normal	2 (40)	0 (0)				
	Abnormal	3 (60)	3 (100)				

^a^SVV: subjective visual vertical.

^b^cVEMP: cervical vestibular evoked myogenic potential.

^c^oVEMP: ocular vestibular evoked myogenic potential.

^d^OR: odds ratio.

When examining cVEMP, among those with normal cVEMP responses, 3 (38%) had normal SVV values, while 5 (63%) had abnormal SVV values. Among those with abnormal cVEMP responses, 4 (80%) had abnormal SVV values. The *P* value for this association was >.99, indicating no statistically significant relationship between cVEMP and SVV values. However, the OR was 2.40 (95% CI 0.18-32.88), suggesting a higher likelihood of abnormal SVV values among those with abnormal cVEMP responses, although this was not statistically significant. Similarly, when analyzing oVEMP, among those with normal oVEMP responses, 2 (40%) participants had normal SVV values, while 3 (60%) had abnormal SVV values. Among those with abnormal oVEMP responses, all patients (100%) had abnormal SVV values. The *P* value for this association was .46, indicating no statistically significant relationship between oVEMP and SVV values. The OR was 2.00 (95% CI 0.90-4.45), suggesting a trend toward abnormal SVV values among those with abnormal oVEMP responses, though it was not statistically significant.

A total of 4 had abnormal SVV and cVEM*P* values, 2 of whom showed deviation to the affected side of the SVV and the abnormal side of the cVEMP. Also, 3 had abnormal SVV and oVEM*P* values, all of whom showed deviation to the affected side of the SVV and the abnormal side of the oVEMP.

### Correlation of SVV With Age, Sex, Affected Side, and DHI Score

In the correlation between SVV values and both the age and DHI scores of patients, the Spearman correlation coefficient (ρ) between SVV and age was –0.327, suggesting a moderate negative correlation, although the *P* value of .25 indicates that this correlation was not statistically significant. Similarly, the correlation between SVV and DHI scores yielded a Spearman correlation coefficient of 0.077, indicating a moderate positive correlation, but again, the *P* value of .80 indicates that this correlation was not statistically significant.

Table 4 presents the association between SVV values and both the sex and affected side of patients. Among male patients, the mean SVV value was 0.60º (SD 3.02º), while the median SVV was –0.29º (IQR –1.96 to 3.61). For female patients, the mean SVV value was higher at 2.19º (SD 6.95º) and the median SVV was 0.20º (IQR –2.62 to 5.84). The *U* statistic, measuring the difference between male and female SVV values, was 19.00 (*P*=.70), indicating no statistically significant difference in SVV between male and female patients. Regarding the affected side of the patients, those with right-sided conditions exhibited a higher mean SVV value of 3.09º, compared to –0.33º for left-sided conditions. The median SVV for right-sided conditions was 0.80º (IQR –0.13 to 7.62), while for left-sided conditions, the median SVV was –0.85º (IQR –3.14 to 2.90). The *U* statistic comparing right and left-sided SVV values was 15.00, (*P*=.28), indicating no statistically significant difference in SVV between patients with right and left-sided conditions.

**Table 4:** Association between SVVa with sex and side of patients (n=14).

Variable		Mean (SD)	Median (IQR)	*U*		*P* value	
**Sex**					19.00		.70
	Male	0.60 (3.02)	–0.29 (–1.96 to 3.61)				
	Female	2.19 (6.95)	0.20 (–2.62 to 5.84)				
**Affected side**					15.00		.28
	Right	3.09 (6.85)	0.80 (–0.13 to 7.62)				
	Left	–0.33 (3.54)	–0.85 (–3.14 to 2.90)				

^a^SVV: subjective visual vertical.

## Discussion

### Principal Results

The reference range for SVV was calculated for 15 healthy participants, including the median value. The mean values and reference ranges were 0.21º (reference range –2.6 to 2.3) for the sitting position. There was no significant correlation between the SVV values and age or sex in the healthy participants. Among the 14 patients with unilateral peripheral vestibular disorder, 6 (43%) had abnormal SVV values. Among the 14 patients, 9 (64%) had abnormal cVEM*P* values, 6 (43%) had abnormal oVEM*P* values, and 12 (86%) had abnormal SVV or VEM*P* values. No significant difference was found between the presence or absence of abnormal SVV values and the presence or absence of abnormal cVEMP and oVEM*P* values; however, the ORs suggested a higher likelihood of abnormal SVV values among those with abnormal cVEMP and oVEMP responses (OR 2.40, 95% CI 0.18-32.88; *P*>.99; OR 2, 95% CI 0.90-4.45; *P*=.46, respectively).

### Comparison With Prior Work

Regarding the SVV reference range, the absolute value of SVV was previously reported to be 2°-3° for static SVV [6,7]; the results of this study are similar to the findings of previous studies. In this study, there was no correlation between age and SVV values, thus indicating that the SVV test was not affected by age and was a stable test with slight variability; Zwergal et al [7] also reported this finding. In contrast, Toupet et al [8] reported that SVV values were smallest in patients aged 20-49 years and greater in patients aged 0-19 years and 80 years or older.

In this study, 6 (43%) of 14 patients with unilateral peripheral vestibular disorder had abnormal SVV. The abnormality detection rate of SVV was comparable to that of oVEMP and lower than that of cVEMP. Although VEMP and SVV are tests that assess the otolithic system, they assess vestibulospinal reflexes and spatial cognition, respectively, and the detection rates vary depending on the pathology and stage of the disease. In this study, 12 patients (12/14, 86%) had abnormal SVV or VEMP, suggesting a higher likelihood of abnormal SVV values among those with abnormal c/oVEMP responses, although this was not statistically significant. SVV, in combination with VEMP, may be used as a screening test for otolithic dysfunction.

### Limitations

This study had some limitations. First, although the sample size was set using G*Power, a multivariable analysis could not be performed because of the small number of cases. Second, the relationship between the abnormal SVV rate and VEMP by disease was unclear. Some previous studies have reported no correlation between SVV and oVEMP [9], while others have reported a significant correlation between SVH and oVEMP test results [10]. Future analysis should be limited to the stage and disease. The advantages of the SVV test using a smartphone app and VR goggles are that the test is inexpensive, quick, and can be performed in an outpatient setting without the need for special equipment.

### Conclusions

In conclusion, our SVV app is currently available for the iPhone and can be used without an internet connection after being downloaded; therefore, it can be used anywhere. The average time required for the test in this study was 5 minutes, thus making it easy to perform during outpatient consultations. Therefore, SVV apps have the potential to make a significant contribution as a screening test for otolith dysfunction in telemedicine. Furthermore, a noteworthy advantage of the newly developed app is that it supports VR and 2D. Thus, by using VR goggles, it is possible to reduce the directional bias caused by inspections when conducting the test in a dark place and against a circular background, similar to that in the bucket test [7].

## Appendix

Multimedia Appendix 1 Normality test results for the study variables.

## Abbreviations

cVEMP: cervical vestibular evoked myogenic potential
DHI: Dizziness Handicap Inventory
oVEMP: ocular vestibular evoked myogenic potential
SVV: subjective visual vertical
VEMP: vestibular evoked myogenic potential
VR: virtual reality

## Data Availability

The data sets generated and analyzed during this study are available from the corresponding author upon reasonable request.
