# Supplementary material for: Development of a Subjective Visual Vertical Test System Using a Smartphone With Virtual Reality Goggles for Screening of Otolithic Dysfunction: Observational Study
Source: JMIR Form Res. 2024 Jun 4;8:e53642. doi: 10.2196/53642 (PMC11185901; doi:10.2196/53642)
Supplement: Multimedia Appendix 1 [file formative_v8i1e53642_app1.pdf]

Normality test results for the study variables. (N=14)

Variables	Kolmogorov -Smirnov			Shapiro-Wilk		
	Statistics	Df	*P* value	Statistics	Df	*P* value
Age	0.110	14	.20	0.975	14	.93
DHI	0.161	13	.20	0.901	13	.14
cVEMP	0.211	14	.09	0.792	14	.004
oVEMP	0.286	14	.003	0.767	14	.002
SVV value	0.239	14	.029	0.864	14	.035

※This is a Multimedia Appendix to a full manuscript published in the J Med Internet Res. For full copyright and citation information see http://dx.doi.org/10.2196/jmir.53642
